# Neurobehavioral features in individuals with Kabuki syndrome

**DOI:** 10.1002/mgg3.348

**Published:** 2018-03-13

**Authors:** Cristina Caciolo, Paolo Alfieri, Giorgia Piccini, Maria Cristina Digilio, Francesca Romana Lepri, Marco Tartaglia, Deny Menghini, Stefano Vicari

**Affiliations:** ^1^ Department of Neuroscience Bambino Gesù Children's Hospital IRCCS Rome Italy; ^2^ Genetics and Rare Diseases Research Division Bambino Gesù Children's Hospital IRCCS Rome Italy

**Keywords:** adaptive behavior, genetic syndromes, language abilities, motor skills

## Abstract

**Background:**

Kabuki syndrome (KS) is a disorder characterized by multiple congenital anomalies affecting development and function of multiple systems. Over the years, researchers have attempted to characterize the neurobehavioral phenotype of KS in cohorts of patients enrolled on the basis of clinical assessment. The availability of molecular testing now allows for recruitment of patients with confirmed KS due to *KMT2D* and *KDM6A*.

**Methods:**

The aims of the present study were to investigate the neuropsychological and behavioral profiles of individuals with molecularly confirmed diagnosis of KS, and determine the extent of heterogeneity occurring in these profiles between individuals with clinical diagnosis of KS with and without mutations in *KMT2D*. We also described performance of our cohort in any neuropsychological domain investigated.

**Results:**

We documented a marked variation in the neuropsychological profile of subjects with clinical diagnosis of KS, even though a relatively homogeneous impairment in linguistic domains and motor skills was observed. No significant difference occurred between mutation‐positive and mutation‐negative groups. Phonological disorders and oromotor dysfunctions were also found, and adaptive functioning was characterized by low performance in daily living and in motor domain.

**Conclusion:**

The present study allowed identification of a distinctive neurobehavioral profile in a cohort of individuals affected by KS with or without molecularly confirmed diagnosis. These findings are expected to help clinicians define more accurately targeted protocols for individualized intervention.

## 1 INTRODUCTION

Kabuki syndrome (KS, MIM#300867 and MIM#147929) is a disorder characterized by multiple congenital anomalies involving the development and function of various organ systems (Armstrong et al., 2005; Bögershausen & Wollnik, 2013). Major clinical features include developmental delay, reduced growth, skeletal abnormalities, various organ malformations, and a distinctive facial gestalt reminiscent of the make‐up of actors in traditional Japanese Kabuki theater (Armstrong et al., 2005; Dentici et al., 2015; Kuroki, Suzuki, Chyo, Hata, & Matsui, 1981; Niikawa, Matsuura, Fukushima, Ohsawa, & Kajii, 1981). In 2010, the molecular cause of KS was identified by Ng et al. (2010) who reported mutations in *KMT2D* (MIM#602113) as the major genetic cause of the disorder. More recently, deletions or mutations of *KDM6A* (MIM#300128) have been reported to underlie a small proportion of affected individuals (Banka et al., 2015; Bögershausen, Bruford, & Wollnik, 2013; Lederer et al., 2012).

Over the years, many studies have contributed to the neurobehavioral characterization of individuals with KS, with particular reference to intellectual, language, and speech abilities. Notably, in these studies enrollment of affected subjects has been based exclusively on clinical criteria (Defloor, van Borsel, Schrander‐Stumpel, & Curfs, 2005; Matsumoto & Niikawa, 2003; Mervis, Becerra, Rowe, Hersh, & Morris, 2005; Niikawa et al., 1988; Upton, Stadter, Landis, & Wulfsberg, 2003; Vaux, Jones, Jones, Schelley, & Hudgins, 2005). Niikawa et al. (1988) reported occurrence of cognitive or developmental delay (from mild to moderate) in approximately 90% of affected individuals, and a similar prevalence of intellectual disability (ID) or developmental delay, ranging from 84% to 87%, has been reported in reviews (Matsumoto & Niikawa, 2003; Wessels, Brooks, Hoogeboom, Niermeijer, & Willems, 2002). Severe ID has been documented in a small number of cases (Ho & Eaves, 1997). Sanz, Lipkin, Rosenbaum, and Mahone (2010) described the neuropsychological development of a child with KS documenting specific deficits in nonverbal skills associated with executive dysfunction in the absence of definitive ID. Considering language abilities, phonological and morphosyntactic deficits, preserved receptive skills and good expressive vocabulary have been reported by Van Lierde, Van Borsel, and Van Cauwenberge (2000), while both receptive (vocabulary and grammar) and expressive deficits have been documented by other authors (Burke & Jones, 1995; Galán‐Gómez et al., 1995). Expressive language was also investigated in a small group of individuals with clinical diagnosis of KS and found to be impaired in all subjects (Defloor et al., 2005). Dysarthria and dyspraxia have also been described as common features in KS (Burke & Jones, 1995; Defloor et al., 2005; Upton et al., 2003; Van Lierde et al., 2000). Other studies have been focused on visually based skills and adaptive functioning in individuals with clinical diagnosis of KS. In particular, Mervis et al. (2005) evaluated 11 affected children and documented that most of them functioned in the range of mild ID, with adaptive behavior in the mildly deficient range. Behavioral problems, such as difficulties in attention and/or hyperactivity–impulsivity, and obsession/anxiety, as well as relative weakness in visuospatial construction abilities, were also documented.

Following the identification of the two disease genes underlying the disorder, first attempts to explore possible genotype–phenotype correlations have been carried out (Banka et al., 2015; Lederer et al., 2012; Lindgren et al., 2013; Morgan et al., 2015; Yang et al., 2016). In subjects with inactivating *KDM6A* mutations and gene deletions, consistent cognitive impairment has been observed. In these studies, behavioral problems, including hyperactivity and attention deficit disorder, have been reported to recur in a small fraction of affected subjects. More recently, genotype–phenotype correlation analyses focused on the nature and frequency of speech and language deficits in a relatively small cohort of patients have documented a heterogeneous pattern of oromotor, speech and linguistic impairment (Morgan et al., 2015). Oromotor deficits and dysarthria have been described as a consistent phenotypic feature in patients with KS, and articulation, phonological development, and receptive and expressive linguistic domains were found affected in the majority of cases included in the study. The authors underlined that the multisystem nature of the disorder, involving neurological, orofacial structural, and hearing and cognitive deficits, probably contributed to speech or language impairment.

The aims of the present study were to investigate the neuropsychological and behavioral profiles of individuals with molecularly confirmed diagnosis of KS and verify whether differ from those of mutation‐negative individuals with clinical features fitting KS.

At this aims, participants underwent a detailed neuropsychological evaluation to investigate cognitive, language, motor, and visuospatial skills, and also behavioral and adaptive aspects were assessed.

## 2 MATERIAL AND METHODS

### 2.1 Ethical compliance

Informed consent was obtained from all parents prior to participation and after receiving a comprehensive description of the study. The study was performed in accordance with the Declaration of Helsinki (1964) and was approved by the local ethical committee of the Bambino Gesù Children's Hospital.

### 2.2 Participants

Seventeen Italian individuals, nine with molecularly confirmed diagnosis of KS (MCKS group, hereafter) (six females, three males) (see Table 1) and eight without bona fide *KMT2D* mutations clinically fitting KS (CFKS group, hereafter) (three females, five males), were recruited at the Medical Genetics and Cytogenetics Department of Bambino Gesù Hospital, Rome. Chronological age (CA) of the MCKS group ranged from 3.8 to 13.8 years (8.7 ± 3.7), while CA of the CFKS group ranged from 2.11 to 21.11 (8.1 ± 5.8). Only one participants had epilepsy (patient six, see Table 1) treated with levetiracetam. As part of this study, all individuals underwent a detailed neurocognitive evaluation.

**Table 1:** Characteristics of MCKS group

Subject	Sex	*KMT2D* nucleotide substitution	*KMT2D* amino acid change	Segregation	Age (years;months)	Hearing problems	Cleft lip and/or palate	Final classification
1	F	c.3318dup	p.Ser1107GlnfsTer7	Father not tested	8;3	−	−	Pathogenic
2	M	c.6595delT	p.Tyr2199IlefsTer65	De novo	13;8	−	−	Pathogenic
3	M	c.12800delC	p.Pro4267LeufsTer10	De novo	12;8	CHL	+	Pathogenic
4	M	c.13450C>T	p.Arg4484Ter	Parents not tested	10;7	−	+	Pathogenic
5	F	c.12725C<G	p.Arg4904Ter	De novo	5;3	−	−	Pathogenic
6	F	c.15641G>A	p.Arg5154Gln	De novo	12;3	CHL	−	Pathogenic
7	F	c.12725C>G	p.Pro4242Arg	De novo	3;8	−	−	Pathogenic
8	F	c.15061C>T	p.Arg5021Ter	Mosaic	7;3	−	−	Pathogenic
9	F	c.15535C>T	p.Arg5179Cys	De novo	4;8	−	−	Pathogenic

F, female; M, male; CHL, conductive hearing loss; +, feature present, −, feature absent.

GenBank reference sequence and version numbers: NM_003482 (*KMT2D*).

### 2.3 Instruments

#### 2.3.1 Cognitive abilities

Cognitive profile was assessed using Leiter International Performance Scale–Revised (Leiter–R) Visualization and Reasoning Battery (Roid & Miller, 1997) for the majority of participants. This test gives a brief intelligent quotient (bIQ). The bIQ appears to be psychometrically sound, with reliability estimates ranging from alphas of 0.88 to 0.90. Concurrent validity tests between the Leiter–R (brief and full Scale IQ) and the Wechsler Intelligence Scale for Children (WISC‐III, Wechsler, 1991) (Performance and Full Scale IQ) on children aged 6–16 years, report correlations of .85 and .86 (Roid, Pomplun, Martin, Naglieri, & Goldstein, 2009).

One participant (patient 14, see Table 3) was assessed using Griffith Mental Developmental Scale–Extended Revised (GMDS–ER) (it. ed. Griffith, 2006), which measures the rate of development of young children.

#### 2.3.2 Language, speech, and oromotor abilities

Language was evaluated with regard to the phonological, lexical, and morphosyntax subdomains. Peabody Picture Vocabulary Test (PPVT) (Dunn et al., 1997) was used to assess lexical comprehension. In this test, the examiner pronounces a word describing one of four pictures shown to the participant and asks him or her to point to or say the number of the picture(s) that the word describes. The total score is converted in lexical quotient (LQ).

Lexical production was assessed using Boston Naming Test (BNT) (Kaplan et al., 1983), in which the patient is asked to tell the examiner the name of each picture and is given about 20 s to respond for each question.

Morphosyntax comprehension was investigated using the Test for Reception of Grammar‐2 (TroG‐2) (Bishop et al., 2003). Each test stimulus is presented in a four picture multiple‐choice format.

Phonological aspects were evaluated through a collection spontaneous utterance. We have referred to the phonological development in the Italian language reported by Bortolini (1995) to determine the presence of delay, disorder, or age appropriate phonology.

Oromotor functions were assessed by a speech therapist based on nonverbal movements, like jaw, mouth, lips, and tongue movements.

#### 2.3.3 Motor skills and visuomotor integration abilities

Motor skills were assessed using Movement Assessment Battery for Children–Second Edition (MABC–2) (Henderson et al., 2007). This test identifies motor impairments using three specific motor area composites: manual dexterity, ball skills, and static and dynamic balance. For participants with severe motor impairment, the MABC‐2 protocol according to mental age (MA) was administered, since the motor deficits prevented them from completing the protocol for CA.

The Beery–Buktenica Developmental Test of Visual–Motor Integration (VMI) (Beery & Buktenica, 1997) measures the extent to which individuals can integrate their visual and motor abilities. The child is asked to copy geometric drawings onto a form. The drawings are presented in the order of increasing difficulty; with the distinct visual perception (VP) and motor coordination (MC) subtests, it is possible to test one skill set while excluding the other.

#### 2.3.4 Adaptive behavior

The Vineland Adaptive Behavior Scale (VABS) (Sparrow, Balla, & Cicchetti, 2003) was used to assess adaptive behavior. The test is designed to measure four domains, including communication, daily living skills, socialization, and motor skills. The interview is conducted with the main caregiver. The results provide information crucial for the diagnosis of various disabilities and speech impairment. From the test it is possible to define the age equivalent of the subject in each domain.

#### 2.3.5 Behavioral measures

The parent‐report version of the Achenbach Behavior Checklist was used to measure problem behavior, by applying the version appropriate for the age of the participant: Child Behavior Checklist 1½–5 (CBCL 1½–5) (Achenbach & Rescorla, 2000) or Child Behavior Checklist 6–18 (CBCL 6–18) (Achenbach & Rescorla, 2001). Finally we used the long form of the Conners Parent Rating Scale–Revised (CPRS‐L) (Conners, 1997) to gather information about attention/hyperactivity behaviors based on reports provided by the parents.

#### 2.3.6 Analyses

The raw scores of cognitive, linguistic, motor, and visuomotor integration measures were converted into standard scores based on the normative data of each task for CA. For adaptive behavior, scores were converted into equivalent ages (e.a.) and percentile (pc); for behavioral measures, scores were converted in *T* scores. The mean score of the group for each measure considered was compared to the mean of the normative data.

Performances of individuals of the MCKS and CFKS groups were compared by means of the Mann–Whitney *U* test. To examine individual differences, results of each participant were compared with the normative data of each task and the percentage of participants showing deficits (≤2 standard deviation (*SD*) below the mean or ≤5th pc) was reported.

## 3 RESULTS

The MCKS and CFKS groups did not differ for CA. Moreover, comparisons between the two groups obtained in neuropsychological and behavioral aspects (see Table 2) did not shown any significant difference in any measure considered (Mann–Whitney *U* test: *p* always >.1).

**Table 2:** Comparison between groups

Neuropsychological evaluation	MCKS group (*M* ± *SD*)	CFKS group (*M* ± *SD*)	*Z* adjusted	*p*‐level^a^
IQ	67 ± 24.9	77.1 ± 24.1	−1.01	.3
PPVT (LQ)	73 ± 7.2	78 ± 9.0	−1.44	.1
BNT (*z*‐score)	−3.4 ± 2.7	−2.5 ± 2.1	−0.69	.5
TroG‐2 (pc)	20 ± 33.4	27 ± 28.4	−0.97	.3
VMI (pc)				
Integration	5 ± 6.8	10 ± 8.1	−1.23	.2
VP	7 ± 12.2	19.4 ± 28.8	−0.77	.4
MC	8 ± 18.2	20 ± 21.8	−0.79	.4
MABC‐2 (pc)				
Manual dexterity	16 ± 18.3	11 ± 14.1	0.59	.5
Ball skills	18 ± 29.1	23 ± 18.8	−0.64	.5
Static and dynamic balance	7 ± 9.1	5 ± 6.1	0.26	.8
VABS (pc)				
Communication	9 ± 14.4	30 ± 26.5	−0.95	.3
Daily living skills	6 ± 13.7	6 ± 10.1	0.11	.9
Socialization	6 ± 10.2	21 ± 21.1	−1.17	.2
Motor skills	2 ± 3.9	2 ± 2.8	0.17	.8
CPRS‐L (*T* score)				
A	50 ± 10.2	65 ± 18.7	−1.61	.1
B	68 ± 16.8	70 ± 26.7	−0.23	.8
C	49 ± 12.6	66 ± 18.2	−1.86	.5
D	44 ± 8.5	54 ± 12.9	−1.62	.1
E	46 ± 6.7	51 ± 20.8	0.12	.9
F	53 ± 15.2	56 ± 19.9	0.00	1.0
G	53 ± 11.4	55 ± 21.3	0.30	.7
H	61 ± 18.8	72 ± 22.4	−0.8	.4
I	53 ± 12.9	70 ± 21.6	−1.77	.1
J	50 ± 13.4	54 ± 12.6	−0.82	.4
K	55 ± 10.1	67 ± 19.6	−1.4	.1
L	64 ± 15.7	68 ± 26.2	−0.3	.7
M	52 ± 15.2	63 ± 18.1	−1.2	.2
N	56 ± 17.1	71 ± 22.7	−1.2	.2
CBCL (*T* score)				
Internalizing	59 ± 10.1	62 ± 12.4	−0.3	.7
Externalizing	53 ± 9.3	61 ± 12.4	−1.0	.3
Total	58 ± 10.9	64 ± 11.4	−1.1	.2

MCKS, molecularly confirmed diagnosis of Kabuki syndrome; CFKS, clinically fitting Kabuki syndrome; IQ, intelligence quotient; LQ, lexical quotient; CPRS‐L, Conners Parent Rating Scale–Revised Long Version; A, oppositional, B, cognitive problems/inattention; C, hyperactivity; D, anxious–shy; E, perfectionism; F, social problems; G, psychosomatic; H, ADHD index; I, CGI restless–impulsive; J, CGI emotional lability; K, CGI total; L, *DSM–IV* inattentive; M, *DSM–IV* hyperactive–impulsive; N, *DSM–IV* total; CBCL, Child Behavior Checklist; PPVT, Peabody Picture Vocabulary Test; BNT, Boston Naming Test; TroG‐2, Test for Reception of Grammar‐2; VMI, Visual–Motor Integration Test; VP, visual perception; MC, motor coordination; MABC‐2, Movement Assessment Battery for Children–Second Edition; VABS, Vineland Adaptive Behavior Scale; pT, *T* score; pc, percentile; *SD*, standard deviation.

a Significant at *p* < .05.

Concerning cognitive profile, seven individuals (41%) obtained a score below 2 *SD* from the mean, four individuals (24%) showed a score lower than 1 *SD* from the mean, and six individuals (35%) obtained a score in average.

Concerning language (Table 3), 15 of 17 participants completed the tasks. In lexical comprehension task, only 13% of the participants obtained a score within 1 *SD* from the mean. Also considering lexical production, a lower percentage of participants, obtained a score in average (14%) and more than half (53%) had a score below 2 *SD* from the mean. In sentence comprehension, the 43% of participants, obtained a score lower than 2 *SD* below the mean; the same percentage obtained a score in average.

**Table 3:** Cognitive, speech, and language abilities, visuomotor, visual perception, motor coordination, motor skills, and adaptive behavior

Subject	IQ/GQ	PPVT LQ	BNT *SD*	TroG‐2 SS	Oromotor functions	Phonological disorder/delay	VMI Int pc	VMI VP pc	VMI MC pc	MABC‐2 pc	VABS							
											Communication		Daily living		Socialization		Motor skills	
											e.a.	pc	e.a.	pc	e.a.	pc	e.a.	pc
1	60	75	−1.7	87	−	−	3	<1	2	5	7.4	47	6.3	9	6.10	9	5.0	1
2	42	69	−8.2	64	+	−	<1	<1	<1	63	9.5	4	7.7	<1	10.1	9	n.e.	n.e.
3	46	72	−6.4	69	+	+	<1	<1	<1	5	9.6	9	7.4	<1	10.7	32	n.e.	n.e.
4	60	65	−2.8	55	−	+	<1	<1	<1	1	6.2	4	5.6	<1	7.1	3	n.e.	n.e.
5	98	74	−0.01	96	+	+	3	7	<1	<1	2.10	<1	4.7	42	2.9	<1	2.9	<1
6	52	64	−4.0	69	+	+	<1	<1	3	5	9.0	7	7.10	4	7.10	1	n.e.	n.e.
7	48	n.e.	n.e.	n.e.	+	+	n.e.	n.e.	n.e.	n.e.	1.8	<1	1.11	<1	2.2	1	1.5	<1
8	89	83	−1.2	71	+	−	16	32	53	9	5.4	7	3.7	<1	3.1	<1	3.5	<1
9	109	83	−2.8	126	+	+	16	21	5	<1	3.4	5	2.9	1	3.10	3	3.5	9
10	103	91	−1.4	104	+	+	23	75	32	1	7.7	50	6.0	23	7.4	50	n.e.	n.e.
11	73	76	−1.4	78	+	+	<1	<1	<1	<1	3.4	3	2.6	<1	4.11	19	2.0	<1
12	91	86	−1.8	67	−	−	12	23	37	9	8.11	55	4.7	3	6.11	32	n.e.	n.e.
13	25	64	−7.0	n.e.	−	+	n.e.	n.e.	n.e.	5	3.2	<1	4.6	<1	3.6	<1	n.e.	n.e.
14	78	n.e.	n.e.	n.e.	n.e.	+	n.e.	n.e.	n.e.	n.e.	n.e.	n.e.	n.e.	n.e.	n.e.	n.e.	n.e.	n.e.
15	98	77	−2.8	105	−	−	16	16	50	25	5.6	50	3.11	<1	5.9	45	3.7	5
16	76	84	−2.7	91	+	+	9	2	4	<1	11	50	8.9	19	7.7	5	n.e.	n.e.
17	73	73	−0.8	73	+	+	5	<1	<1	5	3.7	<1	2.5	<1	2.4	<1	3.0	

IQ, intelligence quotient; GQ, general quotient; PPVT, Peabody Picture Vocabulary Test; BNT, Boston Naming Test; TroG‐2, Test for Reception of Grammar‐2; VMI int, Visual–Motor Integration Test; VP, visual perception; MC, motor coordination; MABC‐2, Movement Assessment Battery for Children–Second Edition; VABS, Vineland Adaptive Behavior Scale; *SD*, standard deviation; pc, percentile; LQ, lexical quotient; SS, standard score; +, presence of difficulties, −, absence of difficulties; n.e., not evaluated; e.a., equivalent age.

Regarding oromotor functions, more than half (69%) of participants had difficulty reproducing the nonverbal movements on imitation. Moreover, 71% of the participants had a phonological disorder (Table 3).

Concerning motor skills, in Global Motor Index Area of the MABC‐2, only two participants (13%) obtained an average score in Global Motor Index Area (Table 3). Regarding results in VMI, more than half of the participants (57%) obtained a score ≤5th pc both in visual–motor integration and in VP subtest. In the MC subtest the percentage of individuals with a score ≤5th pc was 71% (Table 3).

For the adaptive behavior, scores were converted into e.a. and pc. In communication domain of the VABS, the half of the subjects obtained a score ≤5th pc. In daily living and socialization domains, more than half of the participants (75% and 56%, respectively) obtained a score ≤5th pc, while in motor domain, investigated in eight subjects, 86% of these obtained a score ≤5th pc.

For behavioral measures, scores were converted into *T* scores (Table 4). Mean *T* score obtained in internalizing, externalizing, and total scales of the CBCL 1½–5 or 6–18 was in the average range for CA in externalizing problems (*T* score < 60), while in internalizing and total problems the mean was, respectively, 60 and 61. Considering individual differences, clinical scores (*T* score ≥ 70) were found in four participants (26%) in internalizing problem scale, in two participants (13%) in externalizing problem scale, and in four participants (26%) in total problems scale. Considering CPRS‐L, a score below 1 *SD* from the mean (*T* score ≥ 60) was found in more than half (73%) of the participants in the cognitive/attention problem subscale (subscale B), in 67% of the participants in *DSM–IV* attention problem subscale (subscale L), and in 60% of the participants in ADHD index subscale (subscale H).

**Table 4:** Behavioral evaluation

Subject	Behavioral Measures																
	CPRS‐L														CBCL		
	Subscale A	Subscale B	Subscale C	Subscale D	Subscale E	Subscale F	Subscale G	Subscale H	Subscale I	Subscale J	Subscale K	Subscale L	Subscale M	Subscale N	Internalizing problems	Externalizing problems	Total problems
	pT	pT	pT	pT	pT	pT	pT	pT	pT	pT	pT	pT	pT	pT	pT	pT	pT
1	61	69	67	59	52	60	52	66	53	55	54	65	77	74	72	61	71
2	47	68	41	35	42	43	54	49	49	41	46	55	45	49	44	43	43
3	40	64	40	40	40	80	40	40	40	40	40	61	40	40	76	63	73
4	57	54	40	45	51	40	40	51	45	48	45	50	43	49	48	48	49
5	62	91	66	57	48	48	67	95	76	50	69	86	69	79	60	59	58
6	64	92	44	37	43	73	53	63	44	80	57	67	48	56	62	55	63
7	40	40	40	40	40	40	40	40	40	40	65	40	40	40	59	35	47
8	40	62	40	40	40	40	65	68	65	61	66	68	40	40	58	60	69
9	45	80	65	48	59	61	67	83	66	39	57	89	71	81	56	54	57
10	43	66	52	51	35	36	56	57	50	48	50	61	43	53	48	47	53
11	82	99	91	65	44	74	48	99	99	73	94	98	78	98	62	69	71
12	74	75	70	59	61	76	97	79	77	61	75	73	72	75	73	76	74
13	n.e.	n.e.	n.e.	n.e.	n.e.	n.e.	n.e.	n.e.	n.e.	n.e.	n.e.	n.e.	n.e.	n.e.	n.e.	n.e.	n.e.
14	n.e.	n.e.	n.e.	n.e.	n.e.	n.e.	n.e.	n.e.	n.e.	n.e.	n.e.	n.e.	n.e.	n.e.	n.e.	n.e.	n.e.
15	70	40	66	40	40	40	40	65	80	62	78	40	65	66	55	51	51
16	40	40	40	40	40	40	40	40	40	40	40	40	40	40	58	54	59
17	81	100	78	71	90	75	52	93	78	44	70	98	83	94	76	73	78

pT, *T* score; CPRS‐L, Conners Parent Rating Scale–Revised Long Version; A, oppositional; B, cognitive problems/inattention; C, hyperactivity; D, anxious–shy; E, perfectionism; F, social problems; G, psychosomatic; H, ADHD index; I, CGI restless–impulsive; J, CGI emotional lability; K, CGI total; L, *DSM–IV* inattentive; M, *DSM–IV* hyperactive–impulsive; N, *DSM–IV* total; CBCL, Child Behavior Checklist.

## 4 DISCUSSION

The present study is one of the first attempts to systematically investigate the cognitive, neuropsychological, and behavioral profile of individuals with molecularly confirmed diagnosis of KS. Cognitive, neuropsychological, and behavioral assessment was also performed on a second cohort of individuals with clinical diagnosis of KS, but lacking bona fide mutations in *KMT2D*. No significant difference in any neuropsychology and behavior measure was identified indicating that the molecular cause underlying the clinical phenotype in the latter group does not differentially impact on the neuropsychological and behavioral profile.

Our results documented approximately two thirds of the subjects had cognitive impairment. Specifically, the mean nonverbal IQ of participants fell in the borderline range with around a quarter of them obtaining an IQ in the borderline range, and the 41% obtaining a score below 2 *SD* from the mean. Our results partially confirmed the high percentage reported by Matsumoto and Niikawa (2003) that concluded that 84% of individuals with KS had an IQ below 80 with a wide range of variability. Such a discrepancy could be related to the use in our cohort of a brief scale to evaluate participants' cognitive abilities that could in part overestimate the performance. On the other hand, our results were consistent with previous studies reporting severe cognitive impairment in only a small number of affected individuals (Ho & Eaves, 1997; Mervis et al., 2005). Indeed, in our study only one participant had an IQ below 4 *SD* from the mean, indicating that severe ID is not a common finding in KS.

Concerning linguistic abilities, we documented deficits in language domains, both comprehension and production. Indeed, only 13% and 14% of subjects obtained a score within 1 *SD* from the mean in PPVT (lexical comprehension) and BNT (lexical production). In morphosyntactic comprehension (TroG‐2), more than half of participants obtained a score at least below 1 *SD* from the mean. These results were in line with a previous study investigating linguistic abilities in KS, which adopted the same tasks to evaluate linguistic comprehension (i.e., PPVT and TroG), reporting low scores in the two comprehension tasks (Mervis et al., 2005) and a deficit also in the lexical production. Moreover, the present data are consistent with literature reporting heterogeneous results in language and the possibility of a huge variability in linguistic skills of individuals with KS (Defloor et al., 2005; Vaux et al., 2005).

Concerning the nature of linguistic impairment, our hypothesis is in accordance with Morgan et al. (2015) that studied a cohort with molecularly confirmed diagnosis of KS and suggested that linguistic deficit is not the key feature of the syndrome but instead may be the result of the neurological, orofacial structural, hearing, and cognitive deficit in KS. Regarding speech, our results indicated that phonological disorder and oromotor dysfunction characterized more than half of our participants (71% and 69% of cases, respectively). Even if not documented for each participant, the present results were in accordance with previous studies reporting very frequent speech and oromotor deficits in KS (Burke & Jones, 1995; Defloor et al., 2005; Morgan et al., 2015; Upton et al., 2003; Van Lierde et al., 2000).

Considering gross motor skills, our study documented a very high percentage of individuals (87%) that obtained a score between 2 and 1 *SD* below the mean or a score below 2 *SD* from the mean for their MA. These results confirmed previous reports underlining the presence of dyspraxia and considerable weakness in motor skills in KS (Mervis et al., 2005). Also, hypothesized in literature, significant joint laxity and hypotonia may be considered as a crucial factor to explain motor deficits (Matsumoto & Niikawa, 2003; Bögershausen et al., 2013; Bögershausen & Buktenica, 2013). Our results in VMI also confirmed the poor visuomotor integration, visuoperception, and motor coordination abilities found in other studies (Mervis et al., 2005).

In adaptive functioning, our participants achieved higher mean equivalent ages in communication than in daily living skills. These results, consistently with previous studies (Mervis et al., 2005), could be due to motor and visuospatial impairment that interferes with daily living activities requiring fine‐ and gross‐motor skills, such as bathing and dressing.

Finally, considering behavioral aspects, internalizing and externalizing problems were not found in our cohort (pT mean always <70). This finding was in line with those reported by Mervis et al. (2005) that did not document pathological evidence in internalizing, externalizing, and total scales of the CBCL. On the other hand, in the CPRS‐L questionnaire more than half of our participants obtained borderline or clinical scores in scales exploring the presence of attentional deficits. Mild attention deficit and/or hyperactivity in KS were also reported in previous studies (Banka et al., 2015; Lederer et al., 2012; Lindgren et al., 2013; Mervis et al., 2005; Wessels et al., 2002; Yang et al., 2016) and these features are deserving of depth future investigation.

In summary, the present study allowed us to identify a distinct neurobehavioral profile in individuals with KS with peaks and valleys of abilities. Globally neuropsychological profile appeared to be similar in the two groups of mutation‐positive and mutation‐negative cases. Results indicated that KS showed a wide range of IQ, while specific deficits in motor abilities, in linguistic domains, in phonological and oromotor functions were usually present. However, in general, behavioral aspects seemed to be more preserved.

Evaluating distinctive abilities in KS may help clinicians to identify which skills should be targeted for early and individualized intervention. Given the heterogeneous pattern of abilities, it is essential to perform in‐depth evaluations in order to gain a more accurate characterization of the neurobehavioral profile. Specifically, our results suggest that particular interventions should be addressed to strengthen language and motor abilities, which are essential to improve adaptive behaviors and daily living skills in KS. The definition of the neuropsychological and behavioral phenotype in our study allows to emphasize the homogeneity of KS independently from the molecular cause underlying the disorder.

Limitations of the present study include the use of a brief scale to evaluate participants' cognitive abilities, the absence of standardized tasks for evaluating oromotor functions, and the use of the parent report questionnaire to investigate behavioral features with the inherent risk of over‐ or underestimating behavioral problems. Further research is needed using larger sample sizes and gold‐standard research instruments to further support the findings of the present study.

## CONFLICT OF INTEREST

The authors declare no conflict of interest.
